# Hospital Cases—Urinary Calculi in the Male and Female

**Published:** 1866-06

**Authors:** Frank King


ART. III—*Hospital Cases—Urinary Calculi in the Male and Female. Reported by* Frank King, M. D.

The urinary passages frequently become the seat of calcareous deposits, varying in composition, number and size; but generally giving rise to very distressing symptoms, and causing great uneasiness to the patient.

Urine in its normal state holds in solution a number of saline substances, which are deposited in part on cooling; these are liable to become increased in quantity from a variety of causes, as imperfect digestion, acidity of the stomach, an irritated state of the kidneys or bladder with an increased secretion of mucus. If while such an excess of the urinary salts exists, a foreign body should be present in the bladder, thus affording a nucleus for concretion, they would gradually become attached to it, and by the deposition of successive layers form a stone. But in the majority of cases the nucleus is formed in the kidneys themselves, from which it descends along the ureters, to the bladder, causing more or less irritation in its passage, where, if it is retained from any cause, gradually increases, and ultimately produces disturbance. Thus all calculi have a distinct nucleus, either descending from the kidneys or furnished by a foreign body, introduced from without. In

Dr. Miner's private collection there is one specimen which was engrafted on the end of a metallic catheter, introduced after the operation for perineal section, the patient, a lad, feeling so great relief from his former condition, allowed it to remain too long without being removed.

Calcareous disease is much more frequent in some places than in others; its frequency in limestone regions has long been remarked; habit, occupation, climate, food, drink, disease, etc., are supposed to exert some influence on its frequency, but to what extent or in what way, is not definitely known.

*Case 1st—Stone in the Female.*—Females appear less liable to this disease than males, and get relieved with far less difficulty, when it does occur, owing to the shortness, width, and dilatability of the female urethra. But occasionally a calculus is detained in the bladder or passages and forms a nucleus for the deposit of urinary salts, it gradually enlarges and gives rise to the symptoms characteristic of stone in the bladder. M. S., aged 20, was presented at Prof. Taylor's clinic, (Island Hospital,) suffering from symptoms analogous to those of stone. The female urethra being short, wide, and easily dilated, allows small stones to be seized and extracted through it very readily. In this case sponge tent and sea-tangle were used, and the urethra dilated sufficiently, when a small pair of forceps were introduced and a *hair-pin* extracted with a portion of a stone attached to its end; the stone itself was next seized, it being about the size of a filbert, with a deep dentation where the hair-pin had been attached.

*Case 2d—Stone in the Male.*—S. S., a young lad aged 12, emaciated and anxious looking, residing in the country, has suffered for about three years from a frequent desire to pass water, severe pain in doing so, especially as the last few drops are expelled, referred to the point of the penis, and extending down the inner part of the thighs, occasional sudden interruption of the stream, (caused by the stone falling over the opening and obstructing its passage,) an uneasy feeling produced by any rough motion, and walks with his thighs crossed and drawn close together. On sounding the instrument came in contact with what appeared to be a number of stones. Prof. J. R. Wood decided to perform the bi-lateral operation; the incision was carried across about an inch above the anus and the bladder cut with *Wood's bi-sector.* Four large stones were extracted from his bladder; the wound healed kindly, and in three weeks the little fellow was well.

As soon as the stone has been extracted, the bladder should be washed out, to free it from any chips that may have been broken from the stone, the bleeding arrested and the patient placed in bed, with the legs together. The urine at first flows through the opening, but in a few days it begins to take its course through the natural passage; diet should be light and simple, and the treatment antiphlogistic.

